# Molecular Pathway-Based Classification of Ectodermal Dysplasias: First Five-Yearly Update

**DOI:** 10.3390/genes13122327

**Published:** 2022-12-10

**Authors:** Nicolai Peschel, John T. Wright, Maranke I. Koster, Angus J. Clarke, Gianluca Tadini, Mary Fete, Smail Hadj-Rabia, Virginia P. Sybert, Johanna Norderyd, Sigrun Maier-Wohlfart, Timothy J. Fete, Nina Pagnan, Atila F. Visinoni, Holm Schneider

**Affiliations:** 1 Center for Ectodermal Dysplasias, University Hospital Erlangen, 91054 Erlangen, Germany; 2 Department of Pediatrics, Friedrich-Alexander-Universität Erlangen-Nürnberg, 91054 Erlangen, Germany; 3 Division of Pediatric and Public Health, University of North Carolina Adams School of Dentistry, Chapel Hill, NC 27514, USA; 4 Department of Biochemistry and Molecular Biology, Brody School of Medicine at East Carolina University, Greenville, NC 27834, USA; 5 Division of Cancer & Genetics, Institute of Medical Genetics, Cardiff University School of Medicine, Cardiff, CF10 3AT, UK; 6 Center for Inherited Cutaneous Diseases, University of Milan, 20122 Milan, Italy; 7 National Foundation for Ectodermal Dysplasias, Fairview Heights, IL 62208, USA; 8 Department of Dermatology, Reference Centre for Genodermatoses and Rare Skin Disease (MAGEC), Hopital Universitaire Necker-Enfants Malades, Assistance Publique—Hospitals of Paris, University of Paris-Cité, 75743 Paris, France; 9 Division of Medical Genetics, University of Washington School of Medicine, Seattle, WA 98195, USA; 10 CHILD Research Group, Jönköping University, 55111 Jönköping, Sweden; 11 National Oral Disability Centre for Rare Disorders, The Institute for Postgraduate Dental Education, 55453 Jönköping, Sweden; 12 Department of Child Health, University of Missouri, Columbia, MO 65211, USA; 13 Department of Genetics, Federal University of Parana, Curitiba 80060-000, PR, Brazil; 14 School of Health Sciences, Positivo University, Curitiba 81290-000, PR, Brazil

**Keywords:** ectodermal dysplasia, genetics, classification, XLHED, genodermatosis, LRP6

## Abstract

To keep pace with the rapid advancements in molecular genetics and rare diseases research, we have updated the list of ectodermal dysplasias based on the latest classification approach that was adopted in 2017 by an international panel of experts. For this purpose, we searched the databases PubMed and OMIM for the term “ectodermal dysplasia”, referring mainly to changes in the last 5 years. We also tried to obtain information about those diseases on which the last scientific report appeared more than 15 years ago by contacting the authors of the most recent publication. A group of experts, composed of researchers who attended the 8th International Conference on Ectodermal Dysplasias and additional members of the previous classification panel, reviewed the proposed amendments and agreed on a final table listing all 49 currently known ectodermal dysplasias for which the molecular genetic basis has been clarified, including 15 new entities. A newly reported ectodermal dysplasia, linked to the gene *LRP6*, is described here in more detail. These ectodermal dysplasias, in the strict sense, should be distinguished from syndromes with features of ectodermal dysplasia that are related to genes extraneous to the currently known pathways involved in ectodermal development. The latter group consists of 34 syndromes which had been placed on the previous list of ectodermal dysplasias, but most if not all of them could actually be classified elsewhere. This update should streamline the classification of ectodermal dysplasias, provide guidance to the correct diagnosis of rare disease entities, and facilitate the identification of individuals who could benefit from novel treatment options.

## 1. Introduction

The impaired development of tissues derived from the embryonic ectoderm can lead to a broad spectrum of human disorders, including those from the heterogeneous group of ectodermal dysplasias (EDs). Originally proposed by Freire-Maia, EDs are congenital disorders characterized by alterations in two or more structures of ectodermal origin, involving mainly the skin and its appendages (hair, nails or sweat glands) and the teeth [1,2]. The resulting first approach to a classification of EDs was revised in 2017 by an international advisory group. This expert panel pointed out that EDs are genetic conditions affecting the development and/or homeostasis of two or more ectodermal derivatives, including hair, teeth, nails, and certain glands [3]. More than 180 different types of ED have been described so far based on phenotype and, in some cases, their inheritance pattern. [2,4]. Among the diseases listed in OMIM (Online Mendelian Inheritance in Man^®^), the number of EDs was limited to about 100 entities in 2017 [3]. However, it is still not easy for clinicians to diagnose an ED correctly on the basis of the phenotype alone.

Emerging techniques for exome and genome analysis are now enabling a more accurate diagnosis which considers the affected genes and molecular signaling pathways involved. EDs belong to the genodermatoses and can be inherited in an autosomal recessive, autosomal dominant or X-linked manner, with X-linked hypohidrotic ED (XLHED) being the most frequently diagnosed disorder. The prevalence of hypohidrotic ED (HED) was calculated to be approximately 20 per 100,000 overall, and circa 2 per 100,000 when restricted to molecularly confirmed XLHED [5]. Other EDs are ultra-rare, as reflected by the missing prevalence data. It should, however, also be mentioned that the phenotype of EDs can be rather mild. A portion of such diseases may remain undiagnosed or be classified as non-syndromic disorders of the skin appendages and, thus, the disease per se may be underdiagnosed and its prevalence may be underestimated. Since the publication of the human genome in April 2003, more and more genes with impacts on the development of the embryonic ectoderm have been discovered. This has led to the identification of numerous variants of these genes that can cause ED. 

The first gene discovered as being linked to ED was *EDA*, an X-chromosomal gene encoding the signaling protein ectodysplasin A1 (EDA1) [6]. This is not surprising, since *EDA* variants are responsible for the majority of HED phenotypes [7]. Additional variants of genes have been found in phenotypically similar EDs, e.g., *EDAR*, coding for the EDA1 receptor, and *EDARADD*, encoding the adaptor protein “EDAR-associated death domain” [8]. The discovery of these and other genes underlying ED pointed to different molecular pathways with major roles in the development of ectodermal derivatives, e.g., the EDA/NF-κB, Wnt/β-catenin or the p63 transcription factor pathways. These pathways do not function independently of one another but are linked. With today’s knowledge of the molecular bases of EDs, the classification of the disorders can be expanded to include the involved genes and pathways for a clearer diagnosis. The molecular classification not only assists the clinician in considering differential diagnoses, but also in furthering ED-related research [3]. With drug treatment for XLHED on the horizon, making the right diagnosis is increasingly important [9]. 

This article tabulates all currently known EDs, listed by consensus at a meeting of an international advisory group in June 2022 (Paris). Several new entities are now included, as whole genome/exome sequencing has revealed the molecular basis of many EDs in the last 5 years. It is obvious that there are “blind spots” also for these genetic tests, the current list of conditions will need to be updated regularly based on new information that becomes available in the meantime. Conversely, some previously included syndromes have been excluded because they were defined only by individual case reports or were described more than 20 years ago without molecular diagnosis and, due to a lack of direct contact with the patients at that time or the loss of contact in the interim, cannot be elucidated genetically today. The expert panel also agreed on slightly modifying the current definition of an ED. Collectively, this reduced the number of distinct ED entities from 97 to 49 and made a large step closer towards providing a diagnostic guide to EDs for both clinicians and researchers.

## 2. Classification of Ectodermal Dysplasias and Their Correct Diagnosis

In the latest approach to a clinical, phenotype-based classification of EDs, the relevant disease entities were divided up into 11 subgroups according to the structures affected [2]. For example, the first subgroup comprised 38 diseases with a phenotype in hair, teeth, nails, and sweat glands. The individual disorders differed in their inheritance, e.g., autosomal dominant hypohidrotic ED (ADHED), autosomal recessive hypohidrotic ED (ARHED), or XLHED, and in various additional characteristics (e.g., ectrodactyly, ED and cleft lip/palate syndrome) [2]. A revision of this classification in 2017 by an international expert panel focused on the organization of EDs based on the molecular pathways affected. Disease-causing variants of genes that act along the same molecular pathway were grouped together. That was even the case if the clinical phenotypes and affected structures differed significantly from each other. For example, the EDA/NF-κB pathway comprises genes like *EDA* (OMIM *300451) and *IKBKG* (OMIM *300248). Pathogenic variants of *EDA* cause hypohidrotic ectodermal dysplasia (OMIM #305100), characterized by hypohidrosis, hypotrichosis, hypodontia, and dry skin. On the other hand, mutated *IKBKG*, which is causative of Incontinentia pigmenti (OMIM #308300), leads to short stature, abnormal eye development, hypodontia, and may also affect the central nervous system [3]. This is probably due to the fact that the gene product of *IKBKG* is not only downstream of EDA/EDAR but also of many other ligand/receptor combinations.

The large number of different EDs, however, still makes it difficult for clinicians and researchers to establish a correct diagnosis. Complicating matters further, the expression and the intensity of symptoms vary widely, even within the same disease entity, and some gene variants are only incompletely penetrant. Accurate diagnosis and classification of EDs may help to predict disease severity and is important for the genetic counseling of families. A better quality of life for the patient can be achieved via long-term monitoring and prevention of severe complications. In some countries, a correct diagnosis can also mitigate the enormous socio-economic burden associated with an ED through, for example, adequate dental insurance [10]. Finally, diagnostic accuracy is a pre-requisite for the further characterization of disease entities or the enrolment of patients in clinical trials. 

For these reasons, a five-yearly update of the ED classification was planned. A group of clinicians and scientists, who met at the 8th International Conference on Ectodermal Dysplasias, and additional members of the previous expert panel agreed on conditions for this, namely, (1) to include only those disease entities in a “core ED classification” for which the genetic basis has been clarified and (2) to define the term ectodermal dysplasia more precisely by adding again the attribute “congenital” to its definition. The latter helps to distinguish acquired abnormalities, e.g., nail disorders that appear later in life as a result of trauma or infection, from malformations caused by defective ectodermal signaling. EDs are now described as congenital genetic conditions affecting the development of two or more ectodermal derivatives, including hair, teeth, nails, and certain glands. Table 1 thus comprises the currently known EDs in the strict sense, with the addition of 15 new entities (marked in bold letters) that were not mentioned in the previous classification paper. They have been grouped according to the molecular pathways impaired by the genetic variants, and the affected anatomical structures are indicated. Table 2 displays syndromes with features of ectodermal dysplasia that are related to genes apparently not involved in the already known pathways, in particular the EDA/NF-κB, Wnt/β-catenin, or p63 signaling cascades, and that affect mainly organs of non-ectodermal origin. This applies also to conditions where ED is just a small part of a complex syndrome. Only syndromes included in the most recent ED classification [3] were listed.

## 3. Phenotype

As this classification of the EDs also refers to their phenotype, the characteristics of the affected tissues and anatomical structures will be discussed in more detail in the next section. ED-associated abnormalities of embryonic and fetal development lead to phenotypic traits in two or more of the following tissues: hair, teeth, nails, certain glands (e.g., sweat glands), and other structures derived from the ectoderm.

### 3.1. Glandular Phenotypes

Patients with HED, the most frequent form of ED, have fewer sweat glands than normal, if any. They often present in infancy with unexplained fever (hyperthermia) due to their reduced ability to sweat, a factor which may lead to febrile seizures and even permanent brain injury in some cases. This also applies to fever related to infections or childhood illnesses [16]. Insufficient temperature regulation can be life-threatening and is one of the major contributors to ED-associated mortality [17]. ED patients are expected to suffer more severely from the changing climate than other individuals. Clinical investigations of the presence of sweat glands and sweating can be performed by using a starch–iodine test [18], assessing the sweat pore density crudely with a hand lens or determining it more precisely with a confocal microscope, and/or measuring the pilocarpine-induced sweat production or the skin conductance (before and after stimulation) [19]. This is often neglected in clinical practice, where only nonspecific heat intolerance is noted. The proper quantification of a reported hypohidrosis clearly facilitates differential diagnosis.

Apart from sweat glands, other eccrine glands of ectodermal origin can also malfunction or be absent like the lacrimal, salivary, mammary, sebaceous, and meibomian glands [20]. Disorders of the tear film related to missing meibomian glands and underdeveloped lacrimal glands often lead to dry eyes and may be investigated, for example, with a Schirmer test [21]. Reduced numbers or activity of mucous glands in the upper airways have impact on ciliary function. Decreased secretion of salivary glands can cause a dry mouth and nutritional problems, alter the quality of the voice [22], or increase the susceptibility to opportunistic oral infections [23]. Abnormalities of the mammary glands are associated with impaired breastfeeding in mothers with HED [24], even in heterozygous carriers of an *EDA* variant.

### 3.2. Hair Phenotypes

ED patients often show fine, light, sparse hair that grows slowly or breaks easily. Alopecia universalis is rare, but early balding may occur. Eyebrows and eyelashes are also sparse or absent [25]. The hair phenotype can be further investigated by trichoscopy and light microscopy [26]. Findings in ED patients may include trichorrhexis nodosa and abnormal hair shaft pigmentation [27].

### 3.3. Dental Phenotypes

The oral manifestations of ED range from mild hypodontia to anodontia. The tooth shape may be abnormal, including conical, often pointed teeth, bulbous roots, and/or taurodontism. Teeth may be relatively small, widely spaced and susceptible to mechanical damage [28]. It must be noted that not all ED patients suffer from a congenital lack of teeth but rather sometimes only from less obvious dental problems, such as enamel hypoplasia. Variants of the gene *DLX3* should be mentioned here, which cause tricho–dento–osseous syndrome (TDO, OMIM #190320) [29]. Malformed front teeth might be the first sign of ED in infants. Delayed tooth eruption should also prompt further evaluations for ED [28]. It is not uncommon that an ED is first diagnosed by the dentist based on abnormal tooth development and missing teeth. Dental ED manifestations may result in difficulty chewing and swallowing or in speech problems, for which multidisciplinary care is required.

### 3.4. Nail Phenotypes

Nails can be normal or show mild abnormalities, e.g., hyperconvexity, discoloration or fragility (brittle nail plates, underdeveloped cuticles). In more severe cases, nails are hypoplastic, dystrophic or absent [30,31]. It should be noted, however, that the nail phenotype is often neglected by the examining clinician and/or poorly described in papers and case reports.

### 3.5. Other Manifestations

**Figure 1:**
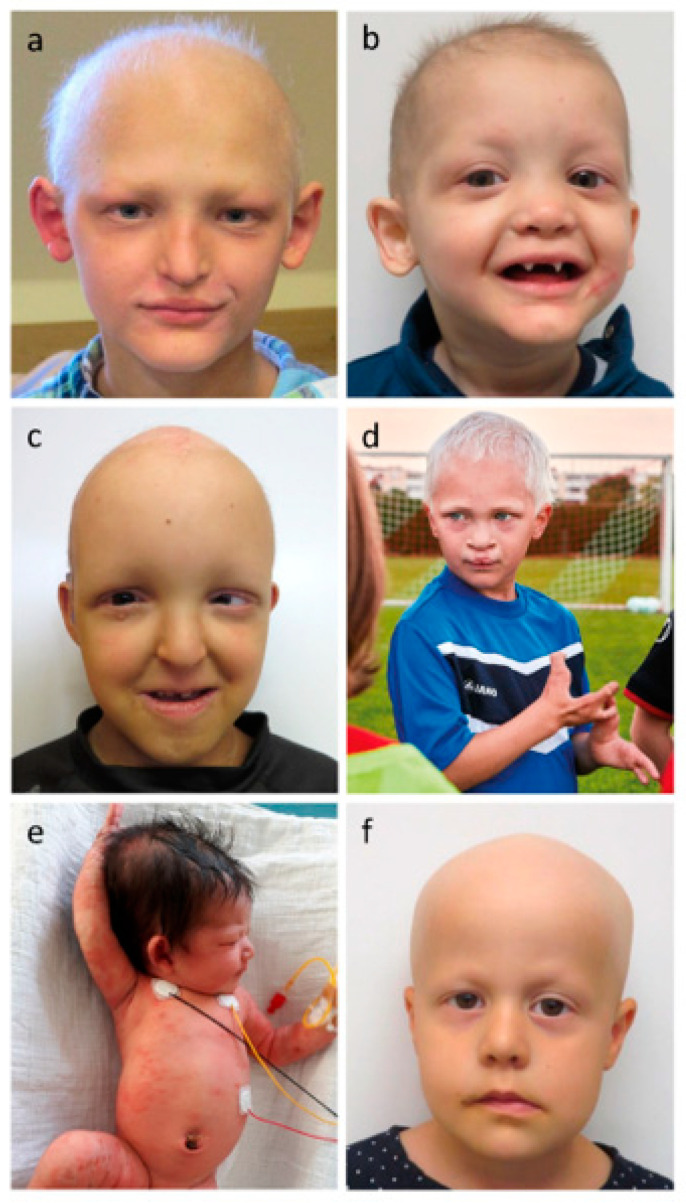
(**a**,**b**) Hypohidrotic ectodermal dysplasia due to pathogenic *EDA* variants (XLHED). Note the sparse hair, missing eyebrows, and midface hypoplasia, indicating the absence of many teeth. (**c**,**d**) Ankyloblepharon–ectodermal dysplasia–cleft lip/palate (AEC) syndrome and ectrodactyly ectodermal dysplasia–cleft lip/palate (EEC) syndrome, respectively, both caused by pathogenic *TP63* variants; (**e**) Incontinentia pigmenti (erythematous, vesicular rash and wart-like skin papules overlapping with the initial vesicular stage, no hyperpigmentation yet) in a newborn infant carrying heterozygously a pathogenic *IKBKG* variant; (**f**) Clouston syndrome (total alopecia, very sparse eyebrows and eyelashes), resulting from pathogenic variants of *GJB6*. All photos unpublished to date.

Further frequent findings in patients with ED include facial characteristics, such as a midface hypoplasia leading to frontal bossing, a saddle nose, thick everted lips, and orofacial clefts, skin abnormalities like palmoplantar hyperkeratosis, very dry skin with poorly developed dermal ridges, an erythematous or scaly skin in newborn patients, periorbital hyperpigmentation, increased skin fragility, chronic erosions, and very often atopic dermatitis [31]. Figure 1 displays the characteristic features of six pediatric patients affected by different forms of ED.

## 4. Molecular Signaling Pathways

The OMIM-listed EDs in Table 1 were classified according to their involvement in the distinct signaling pathways. In addition to the EDA/NF-κB pathway, the p63 transcription factor pathway, and the Wnt/β-catenin pathway, we included a group of genes that play a role in the development of cell structure or keratinization, and, as a fifth group, EDs related to other pathways, such as Notch signaling (e.g., *TSPEAR* variants). There is, however, some overlap, as certain gene products may impact both cell structure and function as the mediator of one of the signaling pathways. For example, cadherin-3 defects due to pathogenic *CDH3* variants could be included both in the structure group and as a target of p63 in its pathway [32]. If the link to a signaling pathway was obvious, we preferred classification in the respective pathway group rather than in the structure group. The phenotype of a patient often allows a rough estimation of the underlying genetic defect, e.g., HED is mainly caused by genes belonging to the EDA/NF-κB pathway [33], while certain dental findings together with nail abnormalities indicate a defect in the Wnt pathway [34].

### 4.1. EDA/NF-κB Pathway

Pathogenic variants of genes related to the EDA/NF-κB pathway can cause either ED or non-syndromic tooth agenesis. The ligand of this signaling pathway is EDA, a homotrimeric type II transmembrane protein cleaved by the protease furin. The gene *EDA* is transcribed and spliced alternatively, generating several isoforms of the protein, the most relevant of which are EDA1 and EDA2 [35]. EDA1 binds to the EDA receptor (EDAR), recruiting the intracellular adapter protein EDARADD, which activates downstream signaling. EDA2, on the other hand, interacts with the EDA2 receptor (EDA2R, formerly known as XEDAR). EDARADD further builds a complex with TNF-receptor-associated factor 6 (TRAF6), TAK1-binding protein 2 (TAB2) and TGF-β-activated kinase 1 (TAK1) [36]. This complex activates the inhibitor of nuclear factor kappa-B kinase (IKK) complex (directly or indirectly). Activation of the IKK complex, which is composed of conserved helix–loop–helix ubiquitous kinase (CHUK, also known as IKK-α) and IKK-β, and a structural component (NEMO/IKK-γ), leads to phosphorylation, ubiquitination, and proteasomal degradation of the NF-κB inhibitor (IkB). Thus, the transcription factor NF-κB is released and translocated into the nucleus where it activates the transcription of a variety of target genes involved in the development and morphogenesis of anatomical structures like teeth, hair, nails, and eccrine glands [36]. The termination of the signaling is possibly achieved by CYLD lysine-63 deubiquitinase which deubiquitinates the TRAF6 protein [37]. The EDA2-EDA2R interaction induces NF-κB activation via the non-canonical EDA/NF-κB pathway [38], but whether pathogenic *EDA2R* (*XEDAR*) variants can cause HED is still under investigation [33].

### 4.2. Wnt/β-Catenin Pathway

The canonical Wnt pathway plays a crucial role in regulating the differentiation, proliferation, and migration of cells, for example during dental and orofacial development. Pathogenic variants of genes involved in this pathway are known to cause either non-syndromic tooth agenesis, hypohidrotic (or hidrotic) ED, or Goltz syndrome [39]. In humans, 19 different Wnt ligands have been described, of which Wnt-10A and Wnt-10B are clinically the most important ones for tooth development [34].

The binding of a Wnt ligand to a transmembrane receptor of the Frizzled receptor family transmits the biological signal to the Dishevelled protein inside the cell. The co-receptors of Frizzled, LDL receptor-related protein (LRP) and Kringle-containing transmembrane protein 1 (KREMEN1), are also involved in tooth development. In the cytoplasm, the activation of canonical Wnt signaling leads to β-catenin accumulation. This protein can now enter the nucleus and act as a coactivator of transcription factors belonging to the T-cell factor/lymphoid enhancer factor (TCF/LEF) family [39]. LEF1, in turn, activates the transcription of EDA, linking Wnt signaling to the EDA pathway [13].

### 4.3. p63 Signaling

**Figure 2:**
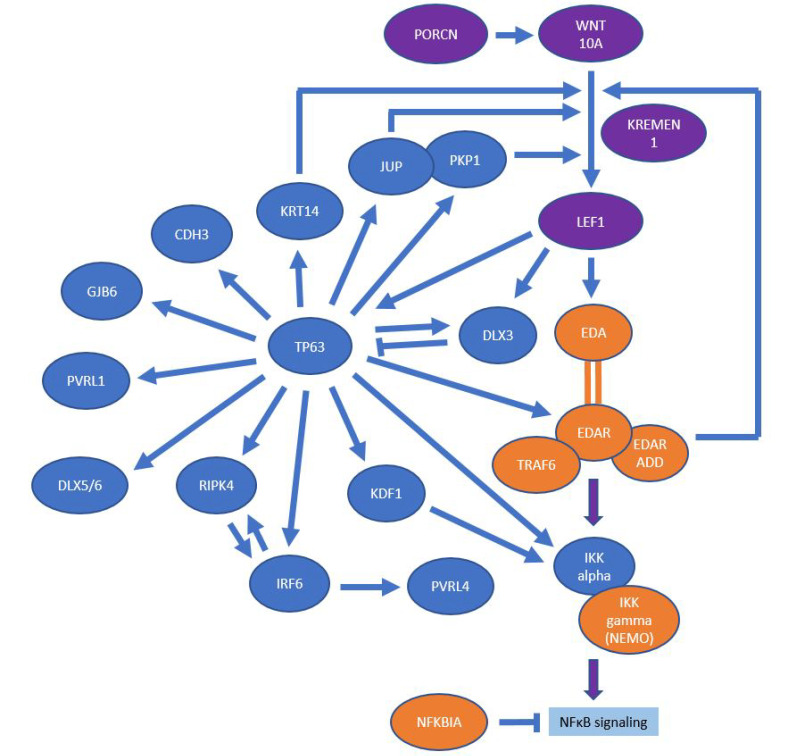
The multiple interactions between the EDA/NF-κB, Wnt and p63 pathways (in orange, purple, and blue, respectively). Arrows indicate stimulatory protein–protein or protein–gene regulatory interactions, bars are used for inhibitory interactions.

Tumor protein p63 is an ancient member of the p53 family of transcription factors. Various human developmental disorders caused by alterations in p63 signaling clearly demonstrate the importance of p63 in the development of the skin and its appendages [40]. The establishment of epidermal fate is controlled by p63 through the regulation of numerous cell activities. A crucial role of p63 as a master regulator in epidermal development has been demonstrated, through which p63 alters the genomic landscape and gene expression of several hundred genes that contribute to human disease. Tumor protein p63 induces directly or indirectly receptor-interacting serine/threonine kinase 4 (RIPK4) [41] or EDAR [42]. The multiple interactions between the EDA, Wnt and p63 pathways are displayed in Figure 2.

### 4.4. Keratinization and Structure-Giving Proteins

Not all proteins important for the normal development of ectodermal derivatives are found in signaling pathways. Another group with similar functions includes proteins with roles in cell structure formation, keratinization, or cell–cell interaction. Keratins are intermediate filament proteins and are essential for providing structural stability to hair, epidermis, and nails [43]. Gap junctions, desmosomes or adherens junctions represent structures that connect cells. Pathogenic variants of genes involved in cell–cell connection/communication are also involved in disorders of ectodermal development [44]. Thus, alterations in connexins, nectins, cadherins, desmoplakin or plakoglobin can be responsible for EDs [45].

## 5. Selection of Diseases to Be Classified

### 5.1. Exclusions

Many of the OMIM-listed EDs were described in the last century only based on their clinical phenotypes, and molecular diagnostics was not performed. It is well possible, however, that recently discovered variants of ED-related genes also caused diseases reported in the last century. This is why we have tried to investigate disease entries retrospectively whose genetic background had not been elucidated by the time of reporting and, if necessary, have reclassified those diseases or removed them from the list of EDs to be classified. We identified a total of 43 diseases in OMIM that were listed as EDs without any report about causative gene variants. For the majority of these EDs, no more than three publications were available, the last of which was usually older than 15 years. For example, two reports about deafness with anhidrotic ectodermal dysplasia (OMIM #125050) have been published, the most recent of which appeared in 1951 [46]. Therefore, we tried to contact the authors of these reports using the contact details indicated in the published paper. Since many of the authors are not alive anymore or retired a long time ago, direct contact could often not be established. In this case, we contacted the institution where the relevant research was conducted. We were able to establish contacts with the authors of 35 OMIM-listed diseases or their institutions and have received 17 responses to the question whether genetic material of the patients would still be available. For six diseases it became clear that genetic material is not available, nor will contact be possible with the patients and/or the authors of the publication. We are continuing this research that may allow an expansion of Table 1 in case of positive findings. The diseases investigated in this way are listed in the Supplementary Material (Table S1).

Ultimately, we decided that we should not classify diseases for which the genetic cause is unknown and only one publication exists, and/or the last publication dated back more than 15 years and/or no response from the authors was received.

### 5.2. New Inclusions

Since the last literature search for the revised ED classification in 2017 [3], several new EDs and their genetic causes have been described. To be mentioned here, for example, variants of *TSPEAR* [47,48], *HOXC13* [49] or *CST6* [50] have been shown to underlie new forms of ED. However, some of these genes have not yet been assigned to ED in the OMIM compendium, i.e., *TRAF6* [11,12], *LEF1* [13,14], or *LRP6* [15]. It is reasonable to assume that the pathogenesis of more EDs will be elucidated at the molecular level in the coming years. Here, we describe a new ED, caused by dysfunctional low-density lipoprotein receptor-related protein 6 (LRP6; encoded by *LRP6*, *OMIM 603507), in more detail to illustrate this point.

Pathogenic variants of *LRP6* have been known so far only as being causative for non-syndromic tooth agenesis (OMIM #616724). LRP6 acts as a Wnt co-receptor and influences the interaction of Wnt and Frizzled, thus activating Wnt signaling. LRP6 is a single transmembrane protein. Its gene belongs to the *LDLR* gene family [51]. The protein has an extracellular domain, a transmembrane portion, and an intracellular part that contains five Pro-Pro-Pro-Ser/Thr-Pro (PPPS/TP) motifs [52]. LRP6 can form a complex with Wnt and Frizzled, leading to phosphorylation of the intracellular region of LRP6 [53] which activates the Wnt signaling cascade.

**Figure 3:**
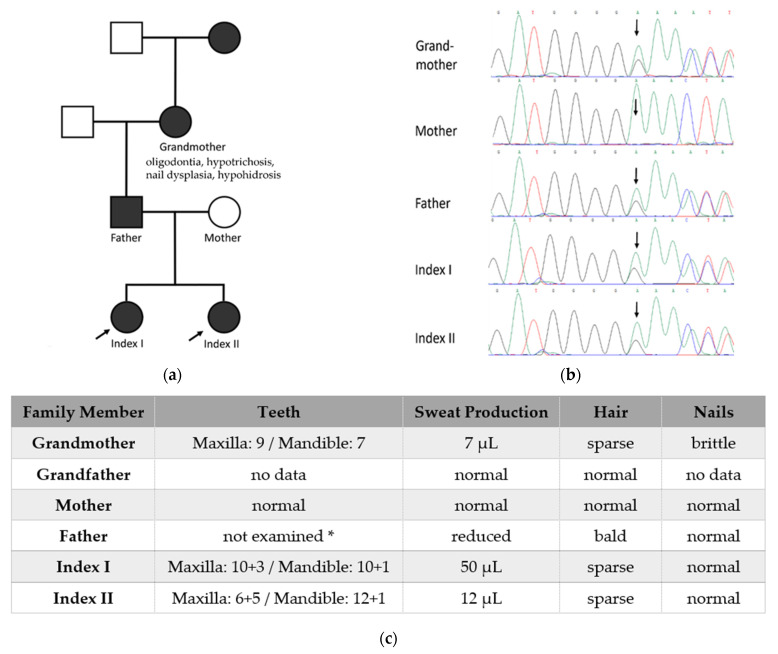
(**a**) Family pedigree showing segregation of the pathogenic *LRP6* variant. Affected male individuals are indicated by black squares, affected females by black circles. Index patient I (arrowed) was subjected to whole exome sequencing; (**b**) Sanger sequencing of the family members’ DNA samples at the mutation site; (**c**) Phenotype in different patients: The number of teeth at the time of radiographic examination is given, the first digit refers to the permanent teeth, the second digit to deciduous teeth. In the grandmother and both index cases, missing permanent dentition was congenital. The ability to sweat is indicated by pilocarpine-induced sweat production within 30 min. * all teeth extracted in childhood.

We studied a non-consanguineous family of German origin with a total of 5 patients (Figure 3a). All affected family members showed more or less pronounced abnormalities of teeth, hair, and sweat glands (Figure 3c). Because family history and pedigree indicated a dominantly inherited defect, we investigated a DNA sample of index patient I by whole exome sequencing. The obtained data set was filtered and analyzed for pathogenic variants of the currently known genes. A heterozygous frameshift mutation, c.4219dupG (p.E1407Gfs*5), was detected on chromosome 12 in *LRP6* (NM_002336.3; NP_002327.2), affecting more than 200 C-terminal amino acids of the LRP6 protein. Sanger sequencing of the mutation site revealed the variant’s heterozygous presence in each of the affected subjects but not in the unaffected family members (Figure 3b). This finding, combined with a recent report of a single case with another *LRP6* mutation [15] that also resulted in symptoms consistent with ED, prompted us to classify *LRP6* as a gene linked to ED and to list it under the Wnt pathway in Table 1.

An explanation as to why mutated *LRP6* led to ED in that family, whereas it used to be linked to non-syndromic tooth agenesis in previous publications [54,55], may arise from considering the molecular function of the protein [53]. The p.E1407Gfs*5 frameshift could result in a truncated protein lacking the intracellular domain that contains phosphorylation sites. This would allow the altered protein to be incorporated into the cell membrane and to interact with its ligand Wnt and its receptor Frizzled. However, as the missing intracellular domain cannot be phosphorylated at its Pro-Pro-Pro-Ser/Thr-Pro (PPPS/TP) motif, the extracellular signal is not transduced and β-catenin is degraded, leading to inactivation of the Wnt pathway. Therefore, the effect of this presumably dominant-negative mutation on early development may be more widespread than that of previously described pathogenic *LRP6* variants [54,55].

## 6. Conclusions

Our first update of the molecular pathway-based ED classification aims at facilitating the clinical and molecular diagnosis of EDs. A five-yearly update can, of course, only be a snapshot that needs to be expanded and improved in the years to come with the help of many researchers, physicians, patients, and patient support groups. This will most likely also speed up the progress we see in managing and treating ED.

## Figures and Tables

**Table 1:** Updated classification of the ectodermal dysplasias with 15 newly added syndromes (bold). * in OMIM only described as isolated tooth agenesis so far ^1^ [11,12]; ^2^ [13,14]; ^3^ [15], (Peschel et al., 2022).

Affected	Disease(s)/Syndrome(s)—Previously Suggested Nomenclature	OMIM No.	Hypo-	Hair	Nail	Glandular	Additional
Gene(s)			dontia	Phenotypic Features			Symptoms
** EDA-NFκB pathway **
	*EDA*	Ectodermal dysplasia 1, hypohidrotic, X-linked (Christ–Siemens–Touraine syndrome, XLHED)	305100	●	●		●	
	*EDAR*	Ectodermal dysplasia 10A and 10B, AD and AR (ECTD10A, B)	129490, 224900	●	●	●	●	
	***EDARADD***	**Ectodermal dysplasia 11A and 11B, AD and AR (ECTD11A, B)**	614940, 614941	●	●	●	●	
	*IKBKG*	Incontinentia pigmenti (IP)	308300	●	●	●		
	*IKBKG*	Ectodermal dysplasia and immunodeficiency 1, AD and AR (EDAID1)	300291, 300301	●	●		●	
	***CHUK***	**Cocoon syndrome**	613630		●	●	●	●
	***NFKBIA***	**Ectodermal dysplasia and immunodeficiency 2 (EDAID2)**	612132	●	●		●	
	***PRKD1***	**Congenital heart defects and ectodermal dysplasia (CHDED)**	617364	●	●	●		●
	***TRAF6***	**Hidrotic form of ectodermal dysplasia, not yet OMIM-listed** ^1^	602355	●	●	●		
**WNT pathway**	*PORCN*	Focal dermal hypoplasia (FDH) or Goltz–Gorlin syndrome	305600	●	●	●	●	●
	*TWIST2*	Focal facial dermal dysplasia 3/**Ablepharon-macrostomia syndrome (AMS)**	136500, 200110		●		●	●
	*WNT10A*	Odonto–onycho–dermal dysplasia (OODD)/Schöpf–Schulz–Passarge syndrome (SSPS)	257980, 224750	●	●	●	●	
	***KREMEN1***	**Ectodermal dysplasia 13, hair/tooth type (ECTD13)**	617392	●	●			
	*TBX3*	Ulnar-mammary syndrome (UMS)	181450	●	●		●	●
	***LEF1***	**Ectodermal dysplasia with or without hypohidrosis, not yet OMIM-listed** ^2^	153245	●	●	●	●	
	***LRP6***	**Ectodermal dysplasia with or without hypohidrosis, not yet OMIM-listed** ^3^	(603507) *	●	●	●	●	
	*MSX1*	Ectodermal dysplasia 3, Witkop type (ECTD3 or Witkop syndrome)	189500	●		●		
**p63 pathway**	*TP63*	Acro–dermato–ungual–lacrimal–tooth syndrome (ADULT)	103285	●	●	●		●
	*TP63*	Ectrodactyly ectodermal dysplasia–cleft lip/palate syndrome (EEC)	604292	●	●	●	●	●
	*TP63*	Limb–mammary syndrome (LMS)	603543	●		●	●	●
	*TP63*	Ankyloblepharon–ectodermal defects–cleft lip/palate (AEC/Rapp–Hodgkin syndrome)	106260, 129400	●	●	●	●	●
	*CDH3*	Ectodermal dysplasia, ectrodactyly, macular dystrophy syndrome (EEMS)	225280	●	●			●
	***KDF1***	**Ectodermal dysplasia 12, hypohidrotic/hair/tooth/nail type (ECTD12)**	617337	●	●	●	●	
	*DLX3*	Tricho–dento–osseous syndrome (TDO)	190320		●	●		
	*RIPK4*	Curly hair–ankyloblepharon–nail dysplasia syndrome (CHANDS);complex lethal subtype known as Bartsocas–Papas syndrome 1 (BPS1)	214350, 263650		●	●		●
**Structure group**	***PKP1***	**Ectodermal dysplasia–skin fragility syndrome (EDSFS)**	604536		●	●	●	●
	***GRHL2***	**Ectodermal dysplasia–short stature syndrome (ECTDS)**	616029	●		●		●
	*PVRL1*	Cleft lip/palate–ectodermal dysplasia syndrome (CLPED1)	225060	●	●	●		●
	*PVRL4*	Ectodermal dysplasia–syndactyly syndrome 1 (EDSS1)	613573	●	●	●		●
	*KRT74*	Ectodermal dysplasia 7, hair/nail type (ECTD7)	614929		●	●		
	*KRT85*	Ectodermal dysplasia 4, hair/nail type (ECTD4)	602032		●	●		
	*GJB2*	Keratitis–ichthyosis–deafness syndrome, AD (KID)	148210		●	●		●
	*GJA1*	Oculo–dento–digital dysplasia (ODDD, ODDR)	164200, 257850	●	●	●	●	●
	*GJB6*	Clouston syndrome or ectodermal dysplasia 2, Clouston type (ECTD2)	129500		●	●		
**Others**	***TSPEAR***	**Ectodermal dysplasia 14, hair/tooth type with or without hypohidrosis (ECTD14)**	618180	●	●	●	●	
	***HOXC13***	**Ectodermal dysplasia 9, hair/nail type (ECTD9)**	614931		●	●		
	***CST6***	**Ectodermal dysplasia 1, hypohidrotic/hair type (ECTD15)**	618535		●		●	
	*AP1B1*	Keratitis–ichthyosis–deafness syndrome, AR (KIDAR)	242150	●	●	●	●	●
	*TRPS1*	Trichorhinophalangeal syndrome, type I (TRPS1) or type III (TRPS3)	190350, 190351	●	●	●		●
	*TRPS1 + EXT1*	Trichorhinophalangeal syndrome, type II (TRPS2)	150230	●	●	●		●

**Table 2:** Syndromes with features of ED that are related to genes apparently not involved in the already known pathways and that mainly affect organs of non-ectodermal origin or where ED is just a small part of a complex syndrome. Only syndromes included in the previous classification of ectodermal dysplasias [3] were listed. Most of them could be classified elsewhere (bold, newly added disease entities). * The genetic abnormality is a duplication in the putative regulatory region (not the promoter) of *SOX9* (located between the *SOX9* and *KCNJ2* genes).

Affected	Syndrome(s) with a Partial Ectodermal Dysplasia-Like Phenotype but Classified Elsewhere	OMIM No.	Hypo-	Hair	Nail	Glandular	Additional
Gene(s)			dontia	Phenotypic Features			Symptoms
CDH1, ***CTNND1***	Blepharocheilodontic syndrome 1/**Blepharocheilodontic syndrome** 2 (BCDS1/**BCDS2**)	119580, 617681	●	●			●
*IFT43, -52,* *-122, -140 WDR19, -35*	Cranioectodermal dysplasia, types 1–4 (CED) or Sensenbrenner syndrome	218330	●	●	●		●
*EVC, EVC2*	Ellis–van Creveld syndrome (EVC)	225500	●	●	●		●
*EVC, EVC2*	Weyers acrofacial dysostosis (WAD)	193530	●		●		●
*KCTD1*	Scalp–ear–nipple syndrome (SENS)	181270	●	●	●	●	●
*SOX9–CNJ2* *	Cooks syndrome	106995		●	●		●
*ANTXR1*	Growth retardation, alopecia, pseudoanodontia, and optic atrophy syndrome (GAPO)	230740	●	●	●		●
***SMARCAD1***	**Huriez syndrome**/Basan syndrome	181600, 129200		●		●	
*DSP*	Carvajal syndrome (DCWHK)	605676	●	●	●		●
*KRT14*	Dermatopathia pigmentosa reticularis (DPR)/Naegeli syndrome (NFJS)	125595, 161000	●	●	●	●	
*KRT16, -17*	Pachyonychia congenita 1/Pachyonychia congenita 2 (PC1/PC2)	167200, 167210	●	●	●	●	
*ARID1A, -1B* *SMARCA4,* *-B1, -E1*	Coffin–Siris syndrome (CSS)	135900	●	●	●		●
*ATP6V1B2*	Deafness, congenital, and onychodystrophy, AD (DDOD)	124480	●		●		●
*TBC1D24*	Deafness, onycho- and osteodystrophy, mental retardation, and seizures syndrome	220500	●		●		●
*SLC25A24*	Gorlin–Chaudhry–Moss syndrome (FPS)	612289	●	●	●		●
*PEX1, **PEX6***	Heimler syndrome 1 (HMLR1)/**Heimler syndrome 2 (HMLR2)**	234580, 616617	●		●		●
*UBR1*	Johanson–Blizzard syndrome (JBS)	243800	●	●			●
*FGFR3, -2 FGF10*	Lacrimo–auriculo–dento–digital syndrome (LADD)	149730	●			●	●
*SREBF1*	Mucoepithelial dysplasia, hereditary (HMD)	158310		●	●		
*HEPHL1*	Pili torti and developmental delay (HJDD)	261990		●	●		●
*KRT81, -83,* *-86, DSG4*	Monilethrix (MNLIX)	158000		●	●		
*RODGI*	Kohlschütter–Tönz syndrome	226750	●	●			●
*INSR*	Pineal hyperplasia, insulin-resistant diabetes mellitus, and somatic abnormalities	262190	●	●		●	●
*CTSK*	Pycnodysostosis (PKND)	265800	●		●		●
*SETBP1*	Schinzel–Giedion midface retraction syndrome (SGS)	269150	●	●	●		●

## Data Availability

Not applicable.

## Supplementary Materials

The following supporting information can be downloaded at: https://www.mdpi.com/article/10.3390/genes13122327/s1, Table S1: Reported disease entities for which the genetic background has not yet been elucidated.
