# Supplementary material for: Molecular Pathway-Based Classification of Ectodermal Dysplasias: First Five-Yearly Update
Source: Genes (Basel). 2022 Dec 10;13(12):2327. doi: 10.3390/genes13122327 (PMC9778228; doi:10.3390/genes13122327)
Supplement: Supplementary file 1 [file genes-13-02327-s001.zip › Supplementary Methods.pdf]

Supplementary Material and Methods:

Patients

A family of German origin was studied over three generations. Grandmother, father, mother and the two daughters have been characterized phenotypically and genotypically. Information about other family members was collected only anamnestically.

DNA Analysis

DNA isolation from peripheral blood, polymerase chain reaction and subsequent Sanger sequencing were performed as described previously [31]. Primers used for Sanger sequencing of *LRP6*: Forward primer TCCGACTGAAGAACCAGCAC, Reverse primer AAGAGGCACAGAAGCTGGTC

Clinical Status

Anthropometric data, HED-relevant issues (including general health, heat intolerance, dentition, skin, nail, and hair abnormalities) were assessed by questionnaires and physical examinations. Tooth status was assessed by questionnaires and panoramic dental x-ray. Quantification of pilocarpine-induced sweating (volumetry) in an area of 57 mm^2 of the forearm for 30 min using the Wescor 3700 device (Wescor, Logan, USA) was performed as described before [9].

Exome Sequencing

EDTA blood sample from one of the sisters (Index I) was sent to a provider of genetic diagnostics and sequencing services (CeGaT GmbH, Tübingen, Germany) for whole-exome sequencing using the Illumina NovaSeq6000 Sequencing Systems. The bioinformatic data obtained were analyzed with the Golden Helix GenomeBrowse tool (Golden Helix,

Bozeman, USA). Criteria for evaluating variants were, among others, population allele frequencies, genomic positions, predicted effects on biological function, and data published in the available scientific literature. Each potentially pathogenic variant identified in this study was assessed with the Ensembl Variant Effect Predictor also containing the SIFT and PolyPhen-2 scores for protein changes (European Molecular Biology Laboratory's European Bioinformatics Institute, Hinxton, UK).
