# Supplementary material for: Molecular Pathway-Based Classification of Ectodermal Dysplasias: First Five-Yearly Update
Source: Genes (Basel). 2022 Dec 10;13(12):2327. doi: 10.3390/genes13122327 (PMC9778228; doi:10.3390/genes13122327)
Supplement: Supplementary file 1 [file genes-13-02327-s001.zip › Table S1.pdf]

Table S1. Reported disease entities for which the genetic background has not yet been elucidated. -, authors contacted repeatedly but no response received; +, contacted and reply received, but still awaiting more detailed information; --, clarified that no more information is available; x, no contact available.

Disease(s) / Syndrome(s)	OMIM No.	OMIM entries	Pubmed entries	Last paper	Authors' reply
Arthritis and interstitial granulomatous dermatitis (Ackerman syndrome)	200970	1	5	2017	-
Acrorenal field defect, ectodermal dysplasia, lipoatrophic diabetes, and other manifestations (AREDYLD) syndrome	207780	2	3	1992	+
Alopecia, contractures, dwarfism, and mental retardation syndrome	203550	4	5	2008	- -
Ameloonychohypohidrotic syndrome	104570	3	2	1975	+
Anonychia-onychodystrophy with brachydactyly type b and ectrodactyly	106990	2	1	1986	+
Anonychia with flexural pigmentation	106750	1	1	1975	+
Arthrogryposis and ectodermal dysplasia	601701	7	4	2010	-
Book syndrome	112300	2	1	1950	- -
Brachymetapody, anodontia, hypotrichosis, and albinoidism	211370	1	1	1968	-
Cataract, hypotrichosis, and mental retardation (CAHMR) syndrome	211770	1	1	1991	-
Cerebellar ataxia and ectodermal dysplasia	212835	5	2	1993	- -
Corneo-dermato-osseous syndrome	122440	3	3	1984	-
Deafness with anhidrotic ectodermal dysplasia	125050	2	2	1951	-
Dermatoosteolysis, Kirghizian type	221810	1	1	1983	-
Dermoodontodysplasia	125640	2	2	1990	-
Ectrodactyly and ectodermal dysplasia without cleft lip/palate	129810	1	1	1988	+
Ectodermal dysplasia, hidrotic, Christianson-Fourie type	601375	1	1	1996	+
Ectodermal dysplasia, hypohidrotic, with hypothyroidism and agenesis of the corpus callosum	225040	3	3	1996	-
Ectodermal dysplasia, hypohidrotic, hypothyroidism and ciliary dyskinesia	225050	4	1	1986	- -
Ectodermal dysplasia, trichoodontoonychial type	129510	2	1	1996	-
Ectodermal dysplasia with adrenal cyst	129550	1	1	1983	-
Ectodermal dysplasia with natal teeth, Turnpenny type	601345	2	1	1995	-
Johnson neuroectodermal syndrome	147770	5	5	2014	- -
Oculotrichodysplasia (OTD)	257960	1	1	1988	-
Odonto-tricho-ungual-digital-palmar syndrome	601957	1	1	1998	-
Odontomicronychial dysplasia	601319	1	1	1996	-
Onychotrichodysplasia and neutropenia	258360	3	1	1991	- -
Pilodental dysplasia with refractive errors	262020	1	1	1985	-
Sener syndrome	606156	4	1	1997	+
Teeth, congenital absence of, with taurodontia and sparse hair	272980	4	1	1973	+
Tetramelic deficiencies, ectodermal dysplasia, deformed ears, and other abnormalities	273400	4	1	1989	+
Thumb deformity and alopecia	188150	3	1	1988	-
Trichodental dysplasia	601453	4	2	1987	+
Trichoodontoonychial dysplasia with bone deficiency	275450	1	1	1983	+
Uncombable hair, retinal pigmentary dystrophy, dental anomalies, and brachydactyly	191482	2	1	1987	-
Ectodermal Dysplasia And Neurosensory Deafness	224800	1	1	1970	x
Ectodermal dysplasia-syndactyly syndrome 2	613576	1	1	2009	x
Polyposis, Skin Pigmentation, Alopecia, And Fingernail Changes	175500	5	5	2012	x
Rosselli-Gulienetti Syndrome	225000	3	3	1994	x
Woolly Hair, Hypotrichosis, Everted Lower Lip, And Outstanding Ears	278200	1	1	1963	x
